# The Three-Dimensional Criteria of Developmental Dysplasia of the Hip Using the Functional Pelvic Plane Is More Useful Than That Using the Anterior Pelvic Plane

**DOI:** 10.3390/jcm13092536

**Published:** 2024-04-26

**Authors:** Shinya Ibuchi, Norio Imai, Yoji Horigome, Hayato Suzuki, Hiroyuki Kawashima

**Affiliations:** 1 Division of Orthopedic Surgery, Department of Regenerative and Transplant Medicine, Niigata University Graduate School of Medical and Dental Sciences, Niigata 951-8510, Japan; ibuchi920shinya@msn.com (S.I.);; 2 Department of Orthopedic Surgery, Uonuma Kikan Hospital, Niigata 949-7302, Japan; 3 Division of Comprehensive Musculoskeletal Medicine, Niigata University Graduate School of Medical and Dental Sciences, Niigata 951-8510, Japan; 4 Department of Orthopedic Surgery, Tachikawa General Hospital, Niigata 940-8621, Japan

**Keywords:** developmental dysplasia of the hip, hip osteoarthritis, anteroposterior plain radiography, computed tomography, 3D image

## Abstract

**Background:** This retrospective cross-sectional study investigated the cutoff values (COVs) for developmental dysplasia of the hip (DDH) using a three-dimensional (3D) pelvic model reconstructed using computed tomography (CT). We included 107 healthy Japanese participants and 73 patients who had undergone curved periacetabular osteotomy (CPO) for DDH between 2012 and 2017. **Methods:** The hip CT images were adjusted to the anterior pelvic plane (APP), functional pelvic plane (FPP), sagittal anterior center-edge angle (ACEA), and sagittal posterior center-edge angle (PCEA). The lateral center-edge angle (LCEA), acetabular roof obliquity (ARO), anterior acetabular sector angle (AASA), and posterior acetabular sector angle (PASA) were measured. Receiver operating characteristic (ROC) curves were used to calculate the COVs, and the association between the parameters was analyzed using multiple logistic regression. **Results:** The ARO (≥10.2°) and LCEA (≤22.2°) were independent influencing factors for the APP, whereas the AASA (≤53.1°) and LCEA (≤24.5°) were independent influencing factors for the FPP. **Conclusions:** The 3D criteria for the diagnosis of DDH in Japanese individuals can identify DDH with insufficient anterior coverage, which anteroposterior plain radiographs cannot visualize, and can help determine indications for acetabular osteotomy.

## 1. Introduction

Developmental dysplasia of the hip (DDH) causes hip osteoarthritis (OA) [1,2] and is the most common cause of coxarthrosis, particularly in Japan and other Asian countries [3,4]. Morphological abnormalities associated with DDH cause cartilage degeneration and osteoarthrosis due to a concentration of joint contact pressure [5,6]. In the diagnosis and assessment of the severity of DDH, the center-edge (CE) angle between a line connecting the center of the femoral head and the acetabular margin and a line perpendicular to a line connecting the bilateral tear drops [7], Sharp angle [8], and acetabular roof obliquity (ARO) [9] from the anteroposterior plain radiograph of the hip joint and, in particular, the lateral CE angle is considered an index of load stress on the acetabular articular cartilage and joint lip [10] and is an important radiographic index. Wiberg defined the CE angle for acetabular dysplasia as normal at ≥25°, borderline at 20–25°, or abnormal at <20° [7]. In Japan, a CE angle < 20°, a Sharp angle > 45°, and an ARO > 15° on anteroposterior plain radiography are used as judgment criteria [3]. However, no consensus currently exists regarding these criteria [11]. Information is available on the lateral coverage of the femoral head obtained from conventional anteroposterior radiographs by measuring the CE angle [7] and anterolateral coverage from false-profile lateral radiographs by measuring the anterior center-edge angle [12,13,14,15]. However, these two-dimensional (2D) images lack three-dimensional (3D) information, and accurate quantification of the degree and location is difficult [16].

In recent years, there has been a growing scientific interest in the use of 3D technologies in orthopedic surgery. Digitalization makes research in orthopedics more accurate and quantitative [17]. The scientific literature [18,19] describes an overview for the 3D technologies and their current applications in orthopedics. In addition, Flaviu et al. apply 3D technologies clinically as a tool for preoperative planning and personalized surgical treatment of tibial plateau fractures [17,20].

This study aimed to determine the cutoff value (COV) of DDH, which distinguishes between symptomatic and asymptomatic hips, by using a 3D pelvic model reconstructed from computed tomography (CT). We also aimed to present the 3D criteria for the diagnosis of DDH.

## 2. Materials and Methods

### 2.1. Participants

For this retrospective cross-sectional study conducted between 1 January 2010 and 31 December 2012, we recruited participants from the families of outpatients and medical staff at our hospital. This study aimed to analyze the hip and knee joint morphology and alignment of the pelvis, hip, and knee to obtain morphological data regarding normal alignment [21]. In total, 9 male (18 joints) and 64 female (128 joints) patients who underwent curved periacetabular osteotomy (CPO) for DDH between 2012 and 2017 were included. Patients who underwent any hip surgery in both the pelvis and femur and had arthritic changes of Tönnis grades 2–3 (20 joints) were excluded.

**Figure 1:**
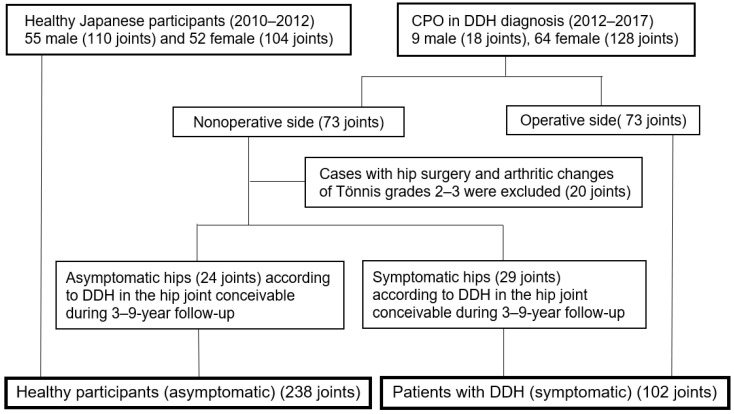
Healthy (asymptomatic) participants and DDH (symptomatic) participants.

Of these 340 joints, patients were divided into two groups: healthy (asymptomatic) (238 joints) and DDH (symptomatic) (102 joints). The healthy group comprised 107 healthy participants (214 joints), from 2010 to 2012, and those with asymptomatic hips (24 joints) on the nonoperative side of the CPO group from 2012 to 2017, according to DDH in the hip joint conceivable during follow-up for 3–9 years. Patients with DDH (symptomatic) (102 joints) were further classified as those with operative side (73 joints) CPO from 2012 to 2017 and with symptomatic hips (29 joints) on the nonoperative side of the CPO group from 2012 to 2017, according to conceivable DDH in the hip joint during the follow-up (Figure 1).

### 2.2. CT

**Figure 2:**
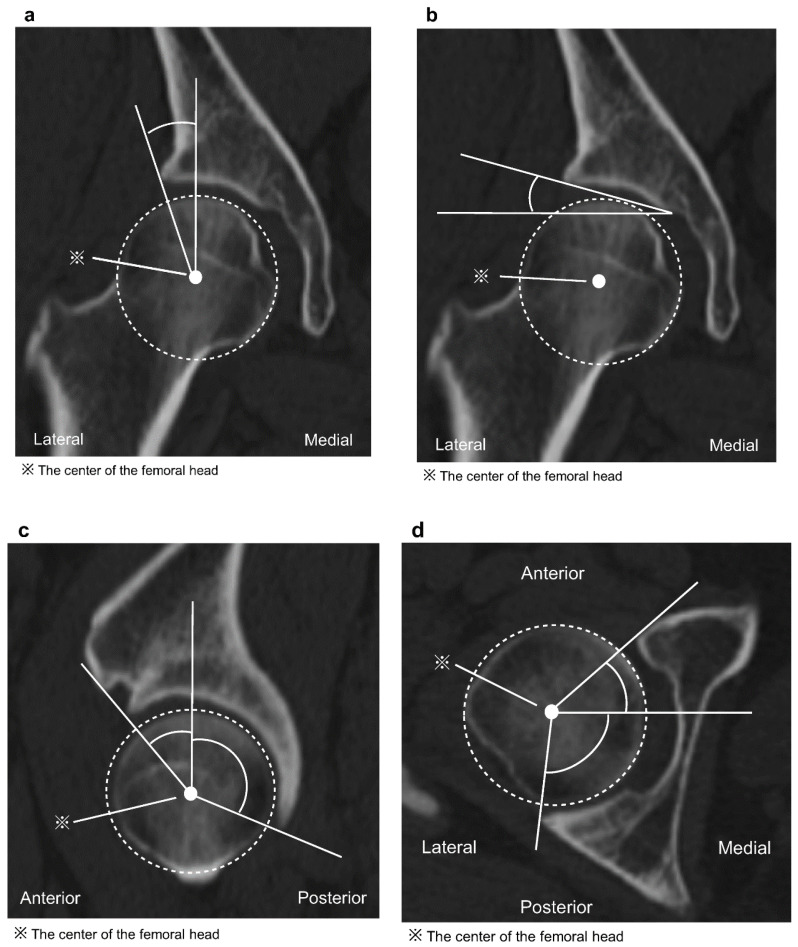
(**a**) The lateral center-edge angle (LCEA) represents the angle between the vertical axis of the pelvis and a line intersecting the center of the femoral head and the lateral acetabular margin. (**b**) The acetabular roof obliquity (ARO) represents the angle between the horizontal axis of the pelvis and a line intersecting the lateral margin of the acetabulum and the superior edge of the fovea. (**c**) The sagittal image of the anterior center-edge angle (ACEA) and posterior center-edge angle (PCEA) represents the angle between the vertical axis of the pelvis and a line intersecting the center of the femoral head and the anterior or posterior acetabular margin. (**d**) The anterior acetabular sector angle (AASA) and posterior acetabular sector angle (PASA) represent the angle between the horizontal axis of the pelvis and a line intersecting the center of the femoral head and the anterior or posterior acetabular margin.

A CT image of the hip joint (1 mm slice from the pelvis to the femoral condyle) with the patient in the supine position was obtained for each patient, with both the hip joint and knee extended and in natural positions. All the hip CTs were taken before the CPO surgery, and these data were reconstructed in 3D using the ZedHip^®^ software (Version 16.0; Lexi, Tokyo, Japan) [22]. First, the pelvis was adjusted to the anterior pelvic plane (APP) reference [23], which is based on the plane consisting of the left and right anterior superior iliac spines (ASISs) and the midpoint of the acetabular node; the sagittal anterior center-edge angle (ACEA) and posterior center-edge angle (PCEA) [24,25] represent the angles between the vertical axis of the pelvis and a line intersecting the center of the femoral head and the anterior or posterior acetabular margin, respectively. The lateral center-edge angle (LCEA) [24,25] represents the angle between the vertical axis of the pelvis and the line intersecting the center of the femoral head and lateral acetabular margin. The ARO [26] represents the angle between the horizontal axis of the pelvis and a line that intersects the lateral margin of the acetabulum and the superior edge of the fovea. The anterior acetabular sector angle (AASA) [27] and posterior acetabular sector angle (PASA) [28], which represent the angles between the horizontal axis of the pelvis and a line intersecting the center of the femoral head, were measured (Figure 2).

**Figure 3:**
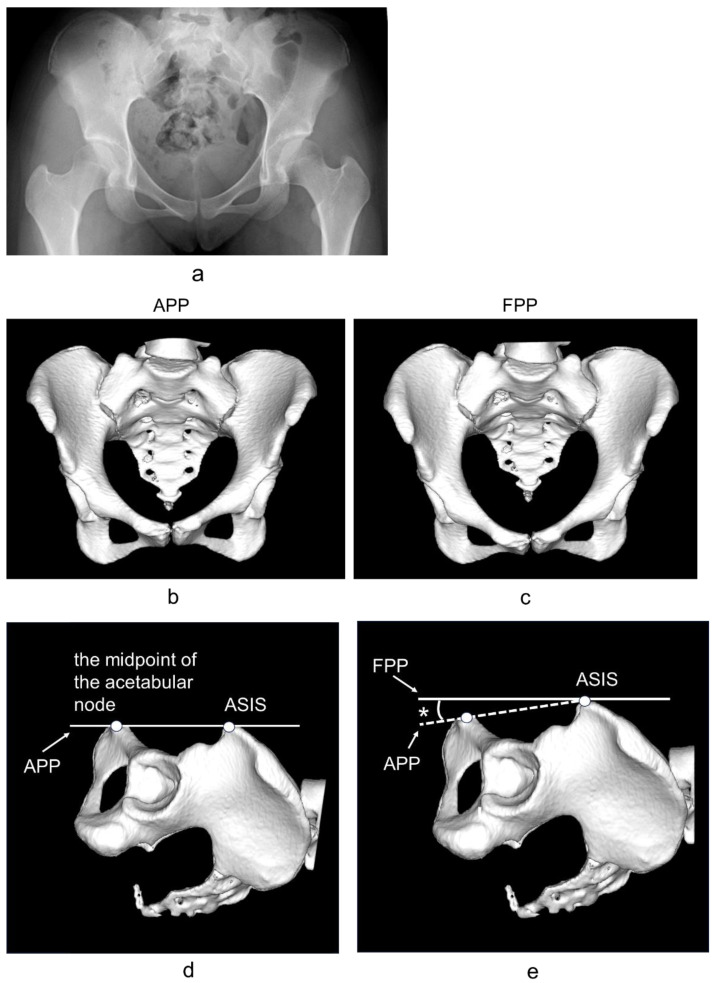
(**a**) Anteroposterior plain radiograph of the hip joint. (**b**) Frontal view of a pelvic three-dimensional computed tomography reconstruction obtained by aligning the pelvis with the anterior pelvic plane (APP), which is based on the plane consisting of the left and right anterior superior iliac spines (ASISs) and the midpoint of the acetabular node. (**c**) Frontal view of a pelvic three-dimensional computed tomography reconstruction obtained by aligning the pelvis with the functional pelvic plane (FPP) (the table plane in the supine position was used as a reference, reflecting the APP sagittal inclination in the supine position). (**d**) Lateral view of a pelvic three-dimensional computed tomography reconstruction obtained by aligning the pelvis with the anterior pelvic plane (APP), which is based on the plane consisting of the left and right anterior superior iliac spines (ASISs) and the midpoint of the acetabular node. (**e**) Lateral view of a pelvic three-dimensional computed tomography reconstruction obtained by aligning the pelvis with the functional pelvic plane (FPP) (the table plane in the supine position was used as a reference, reflecting the APP sagittal inclination in the supine position). * Sagittal inclination of the APP in the supine position.

Additionally, the pelvis was adjusted to the functional pelvic plane (FPP) (the table plane in the supine position was used as a reference, reflecting the APP sagittal inclination in the supine position) [29] and the ACEA, PCEA, LCEA, ARO, AASA, and PASA were measured as in the APP (Figure 3).

These values were measured twice by the two examiners (SI twice and NI once), and the average values were used.

### 2.3. Statistical Analysis

All the statistical analyses were performed using SPSS software version 24 (SPSS Inc, Chicago, IL, USA). Using a receiver operating characteristic (ROC) curve, the COV of the parameter judged to indicate DDH was calculated, and the relationship between the presence or absence of DDH and the ACEA, PCEA, LCEA, ARO, AASA, and PASA (for the APP and FPP) was evaluated to determine the COVs for healthy (asymptomatic) patients (238 joints) and those with DDH (symptomatic) (102 joints). The area under the curve (AUC) of the ACEA, PCEA, LCEA, ARO, AASA, and PASA (for the APP and FPP, respectively) were calculated in the ROC curve.

The COVs of each independent variable judged as DDH in the ROC curve were individually determined, and the relationship between DDH with and without DDH was analyzed using multiple logistic regression. To calculate the dependent variance, the independent variables ACEA, PCEA, LCEA, ARO, AASA, and PASA for the APP and FPP were analyzed using multiple logistic regression without variable selection steps or distributed inflation. Sex, age at the time of CPO, and lifestyle factors were excluded.

To evaluate the validity of the measurement, the intraclass correlation coefficient (ICC) was used for the examiner and between examiners, and the significance was set at *p* < 0.01.

## 3. Results

The average age of the patients at the time of CT imaging was 49.2 ± 29.8 years (range: 20–79 years). The numerical values measured using the APP and FPP are presented in Table 1. In the ROC curve, the COVs for the APP were 49.3° for the ACEA, 22.2° for the LCEA, 10.2° for the ARO, and 51.4° for the AASA. The COVs for the FPP were 52.4° for the ACEA, 24.5° for the LCEA, 10.3° for the ARO, and 53.1° for the AASA. For the PCEA and PASA, the COVs were not calculated for either the APP or FPP (Table 2). The AUC was larger for the FPP for the ACEA, PCEA, LCEA, and AASA, and the APP was larger for the ARO and PASA (Table 3).

In the multiple logistic regression analysis, the ARO and LCEA were independent factors for the APP, whereas the AASA and LCEA were independent factors for the FPP (Table 4). A strong correlation was observed between and within examiners for all the measurement items (ICC > 0.8) (Table 5).

## 4. Discussion

Based on our results, the 3D reference values for Japanese patients with symptomatic DDH were an ARO ≥ 10.2° or LCEA ≤ 22.2° for the APP and an AASA ≤ 53.1° or LCEA ≤ 24.5° for the FPP. Furthermore, the AUCs were larger for the ACEA, PCEA, and LCEA with the FPP. Thus, the FPP may be more useful than the APP for an early diagnosis of symptomatic DDH.

Moreover, an ACEA ≤ 49.3° and AASA ≤ 51.4° for the APP and an ACEA ≤ 52.4° and ARO ≥ 10.3° for the FPP were considered useful COVs for distinguishing between healthy individuals and those with DDH, although they were not significantly influential factors individually. Therefore, considering these factors, only the potential DDH was considered.

DDH is a common disorder of the acetabulum that remains undetected despite childhood screening. Delayed diagnosis or misdiagnosis of DDH can result in the early onset of hip OA and total hip arthroplasty at a young age [30,31,32]. Early detection may facilitate nonsurgical treatment (such as activity modification, nonsteroidal anti-inflammatory drugs, physical therapy, and intra-articular corticosteroid injections), surgical treatment, and follow-up [33].

To detect early-stage DDH, Wiberg defined the CE angle on anteroposterior plain radiographs for acetabular dysplasia as normal at ≥25°, borderline at 20°–25°, and abnormal at <20° [7].

Conversely, Julie et al. [33] reported that the lateral coverage of the acetabulum can be normal, whereas dysplasia can occur anteriorly. Therefore, adding a false-profile view pelvic radiograph to assess the presence of dysplasia anteriorly significantly contributes to both the diagnosis of DDH and the prediction of hip OA. However, identifying the most lateral point of the acetabulum is difficult because it may be affected by the overlap of the anterior edges of the acetabulum and osteophytes, and it is often difficult to determine the DDH in borderline DDH (BDDH) with a CE angle of 20–25°. Vivek et al. [34] reported that the values of the LCEA are consistently inflated on CT relative to plain radiography for a wide variety of hip pathologies, highlighting the need for standardization and validation of CT-based measurements to improve the quality of clinical decision making.

Ito et al. [16] evaluated 84 joints in 55 patients (51 women and 4 men) with DDH (LCE < 20°) in patients with pre- or early OA without radiographic evidence of joint space narrowing, formation of osteophytes or cysts, or deformity of the femoral heads using three-dimensional computed tomography (3DCT). The lateral defect type of DDH in 45 joints (54%) was determined using 2D DDH standard radiography; however, the anterior defect type of DDH in 22 joints (26%) and the posterior defect type of DDH in 17 joints (20%) could not be determined using the 2D DDH standard.

Miyasaka et al. revealed that in 3DCT, the average value of each parameter of the acetabulum in healthy individuals was 58.2° in men and 56.0° in women, the PCEA was 97.1° in men and 102.9° in women, and the LCEA was based on the APP standard of 32.5° in men and 31.6° in women; ARO, 4.7° in men and 5.3° in women; AASA, 61.2° in men and 57.1° in women; and PASA, 94.5° in men and 96.8° in women [35]. The average AASA and PASA of DDH are approximately 35–46°and 80–87°, respectively [16,36]. To the best of our knowledge, this is the first report to present COVs for healthy (symptom-free) hips and DDH using the 3D criteria.

The 3D criteria for the diagnosis of DDH in Japanese individuals can identify DDH with insufficient anterior coverage, which cannot be seen on plain anteroposterior radiographs and can help in the diagnosis of indications for acetabular osteotomy.

However, in this study, both the PCEA and PASA were in the APP, and the FPP tended to be smaller in the DDH group than in healthy hips, although this was not a significant factor in the diagnosis of DDH. Therefore, DDH may not be diagnosed using the PCEA and PASA alone because the PCEA is associated with the LCEA and ACEA.

## 5. Limitations

This study had the following limitations: (1) the population was exclusively Japanese, and (2) the study was retrospective, which could have resulted in a selection bias. The patients were divided according to the presence or absence of symptoms during a follow-up period of three to nine years. This means that the symptoms might have changed over time, and it is impossible to predict whether OA will be associated with DDH in the future. Therefore, the results of this study serve only as criteria for determining whether symptoms appear at an early stage. CT images cannot be adjusted to a standing radiograph, nor can a CT be performed in the standing position. This study only involved 3D evaluation of CT in the supine position, and the 3D criteria for the diagnosis of DDH in the standing position are unknown.

## 6. Conclusions

The 3D criteria for the diagnosis of DDH in Japanese patients were an ARO ≥ 10.2° or LCEA ≤ 22.2° for the APP and an AASA ≤ 53.1° or LCEA ≤ 24.5° for the FPP. The ACEA ≤ 49.3° and AASA ≤ 51.4° for the APP and the ACEA ≤ 52.4° and ARO ≥ 10.3° for the FPP were considered useful for diagnosing “potential DDH”. The 3D criteria for the diagnosis of DDH in Japanese individuals can identify DDH with insufficient anterior coverage, which cannot be seen on plain anteroposterior radiographs and can help in the diagnosis of indications for acetabular osteotomy.

## Figures and Tables

**Table 1:** The ACEA, PCEA, LCEA, ARO, AASA, and PASA were measured by the APP and FPP.

APP	Total (n = 340)	Normal (n = 238)	DDH (n = 102)
ACEA (°) *	50.6 ± 15.2 (−16.9~88.9)	56.9 ± 10.1 (29.6~88.9)	36.7 ± 8.5 (−16.9~60.3)
PCEA (°) *	99.4 ± 16.0 (30.4~136.8)	100.9 ± 14.2 (61.3~136.6)	96.1 ± 14.2 (30.4~136.8)
LCEA (°) *	26.0 ± 4.2 (−13.5~59.8)	32.0 ± 7.8 (0.4~59.8)	13.2 ± 7.4 (−13.5~38.8)
ARO (°) *	10.3 ± 9.1 (−12.5~42.2)	6.3 ± 5.8 (−12.5~36.3)	19.1 ± 5.5 (−12.5~42.2)
AASA (°) *	54.6 ± 10.5 (21.7~82.3)	59.2 ± 8.0 (25.2~82.3)	44.7 ± 6.7 (21.7~62.9)
PASA(°) *	94.6 ± 8.6 (54.1~124.5)	95.8 ± 9.1 (54.1~124.5)	91.8 ± 9.1 (54.1~111.9)
**FPP**	**Total (n = 340)**	**Normal (n = 238)**	**DDH (n = 102)**
ACEA (°) *	54.5 ± 13.7 (−9.4~89.2)	60.1 ± 8.5 (28.1~89.2)	42.4 ± 7.6 (−9.4~61.5)
PCEA (°) *	95.4 ± 16.3 (26.3~142.1)	97.8 ± 14.8 (53.7~142.1)	90.4 ± 14.7 (26.3~128.7)
LCEA (°) *	26.4 ± 12.5 (−13.8~78.5)	32.3 ± 8.6 (7.4~78.5)	13.6 ± 9.2 (−13.8~51.8)
ARO (°) *	9.8 ± 8.8 (−12.4~41.9)	6.0 ± 5.8 (−12.4~32.5)	17.9 ± 5.8 (−12.4~41.9)
AASA (°) *	55.6 ± 10.5 (29.2~80.4)	60.3 ± 7.5 (16.0~63.9)	45.6 ± 7.1 (16.0~80.4)
PASA (°) *	93.0 ± 9.5 (12.1~125.6)	94.5 ± 8.7 (72.5~125.6)	89.7 ± 9.3 (12.1~109.2)

* Mean ± standard deviation (range). ACEA: sagittal anterior center-edge angle, PCEA: sagittal posterior center-edge angle, LCEA: lateral center-edge angle, ARO: acetabular roof obliquity, AASA: anterior acetabular sector angle, and PASA: posterior acetabular sector angle.

**Table 2:** In the ROC curve, the COVs of the parameter were considered for DDH, and the relationships between the presence or absence of DDH and the ACEA, PCEA, LCEA, ARO, AASA, and PASA (for the APP and FPP, respectively) were determined. For the PCEA and PASA, the COVs were not calculated for either the APP or FPP.

APP	COV	Sensitivity	1-Specificity
ACEA	49.3	0.788	0.229
LCEA	22.2	0.931	0.152
ARO	10.2	0.848	0.199
AASA	51.4	0.892	0.210
**FPP**	**COV**	**Sensitivity**	**1-Specificity**
ACEA	52.4	0.844	0.257
LCEA	24.5	0.861	0.067
ARO	10.3	0.781	0.217
AASA	53.1	0.861	0.181

ACEA: sagittal anterior center-edge angle, LCEA: lateral center-edge angle, AASA: anterior acetabular sector angle, and ARO: acetabular roof obliquity.

**Table 3:** The AUC of the ACEA, PCEA, LCEA, ARO, AASA, and PASA (for the APP and FPP, respectively) were calculated in the ROC curve.

	APP			FPP		
	AUC	*p*-Value	95% CI	AUC	*p*-Value	95% CI
ACEA	0.867	<0.001	0.826~0.908	0.880	<0.001	0.841~0.919
PCEA	0.560	0.046	0.502~0.618	0.602	0.004	0.502~0.670
LCEA	0.941	<0.001	0.917~0.965	0.952	<0.001	0.928~0.976
ARO	0.896	<0.001	0.858~0.934	0.876	<0.001	0.835~0.917
AASA	0.868	<0.001	0.830~0.906	0.905	<0.001	0.868~0.942
PASA	0.635	<0.001	0.573~0.695	0.634	<0.001	0.572~0.696

ACEA: sagittal anterior center-edge angle, PCEA: sagittal posterior center-edge angle, LCEA: lateral center-edge angle, ARO: acetabular roof obliquity, AASA: anterior acetabular sector angle, and PASA: posterior acetabular sector angle.

**Table 4:** The COVs of each independent variable judged as DDH in the ROC curve were individually determined, and the relationship between DDH with and without DDH was analyzed using multiple logistic regression. In the multiple logistic regression analysis, the ARO and LCEA were independent factors for the APP, whereas the AASA and LCEA were independent factors for the FPP.

APP	Degrees of Freedom	Odds Ratio	95% CI	*p*-Value
ARO	339	−0.028	−0.031~−0.025	<0.001
LCEA	339	−0.001	−0.014~−0.005	<0.001
**FPP**	**Degrees of Freedom**	**Odds Ratio**	**95% CI**	***p*-Value**
AASA	339	−0.029	−0.032~−0.026	<0.001
LCEA	339	−0.013	−0.017~−0.008	<0.001

ARO, acetabular roof obliquity; LCEA, lateral center-edge angle; and AASA, anterior acetabular sector angle.

**Table 5:** Intraclass correlation coefficient (ICC) was used for the examiner and between the examiners.

APP	Intraobserver			Interobserver		
	MAD (n = 340)	ICC	95% CI	MAD (n = 340)	ICC	95% CI
ACEA *	1.3 ± 1.2 (0.0~5.3)	0.937	0.922~0.949	2.4 ± 1.9 (0.1~7.8)	0.856	0.810~0.901
PCEA *	2.0 ± 1.8 (0.0~7.6)	0.892	0.867~0.912	3.1 ± 2.3 (0.2~8.7)	0.821	0.788~0.854
LCEA *	1.3 ± 1.4 (0.0~6.8)	0.933	0.918~0.946	3.3 ± 2.2 (0.0~8.8)	0.813	0.769~0.849
ARO *	1.1 ± 1.1 (0.0~4.0)	0.966	0.957~0.972	2.4 ± 2.0 (0.1~7.9)	0.854	0.823~0.881
AASA *	0.6 ± 0.5 (0.0~1.9)	0.971	0.964~0.977	1.4 ± 1.5 (0.1~8.8)	0.928	0.918~0.939
PASA *	1.0 ± 0.9 (0.0~5.0)	0.963	0.954~0.970	1.6 ± 1.4 (0.0~8.0)	0.921	0.903~0.937
**FPP**	**Intraobserver**			**Interobserver**		
	**MAD (n = 340)**	**ICC**	**95% CI**	**MAD (n = 340)**	**ICC**	**95% CI**
ACEA *	1.3 ± 1.0 (0.0~4.8)	0.944	0.931~0.954	1.9 ± 12.0 (0.1~7.4)	0.908	0.886~0.925
PCEA *	2.1 ± 1.7 (0.0~5.9)	0.882	0.856~0.908	2.3 ± 1.9 (0.1~6.9)	0.861	0.805~0.913
LCEA *	1.5 ± 1.5 (0.0~7.3)	0.934	0.918~0.946	2.3 ± 2.0 (0.0~7.9)	0.859	0.759~0.919
ARO *	0.9 ± 1.2 (0.0~5.3)	0.952	0.941~0.961	1.8 ± 1.6 (0.0~6.0)	0.909	0.888~0.926
AASA *	1.0 ± 0.7 (0.0~3.0)	0.967	0.959~0.974	1.5 ± 1.2 (0.2~5.0)	0.928	0.912~0.942
PASA *	1.0 ± 0.8 (0.0~3.9)	0.966	0.957~0.972	1.3 ± 1.1 (0.0~4.5)	0.943	0.929~0.954

* *p* < 0.01 was set as the significance level. ACEA: sagittal anterior center-edge angle, PCEA: sagittal posterior center-edge angle, LCEA: lateral center-edge angle, ARO: acetabular roof obliquity, AASA: anterior acetabular sector angle, and PASA: posterior acetabular sector angle.

## Data Availability

All the data generated or analyzed during this study are included in this manuscript.
